# Novel small-molecule SIRT1 inhibitors induce cell death in adult T-cell leukaemia cells

**DOI:** 10.1038/srep11345

**Published:** 2015-06-19

**Authors:** Tomohiro Kozako, Takayoshi Suzuki, Makoto Yoshimitsu, Yuichiro Uchida, Ayako Kuroki, Akiyoshi Aikawa, Shin-ichiro Honda, Naomichi Arima, Shinji Soeda

**Affiliations:** 1 Department of Biochemistry, Faculty of Pharmaceutical Sciences, Fukuoka University, Fukuoka, Japan; 2 Faculty of Medicine, Kyoto Prefectural University of Medicine, Kyoto, Japan; 3 Department of Hematology and Immunology, Kagoshima University Hospital, Kagoshima, Japan; 4 Division of Hematology and Immunology, Center for Chronic Viral Diseases, Graduate School of Medical and Dental Sciences, Kagoshima University, Kagoshima, Japan

## Abstract

Adult T-cell leukaemia/lymphoma (ATL) is an aggressive T-cell malignancy that develops after long-term infection with human T-cell leukaemia virus (HTLV)-1. The identification of new molecular targets for ATL prevention and treatment is desired. SIRT1, a nicotinamide adenine dinucleotide^+^ -dependent histone/protein deacetylase, plays crucial roles in various physiological processes, including aging and apoptosis. We previously reported that ATL patients had significantly higher SIRT1 protein levels than healthy controls. Here, we demonstrate that two novel small-molecule SIRT1 inhibitors, NCO-01/04, reduced cell viability and enhanced apoptotic cells in peripheral blood monocyte cells of patients with acute ATL, which has a poor prognosis. NCO-01/04 also reduced the cell viability with DNA fragmentation, Annexin V-positive cells, and caspase activation. However, a caspase inhibitor did not inhibit this caspase-dependent cell death. NCO-01/04 enhanced the endonuclease G level in the nucleus with loss of the mitochondrial transmembrane potential, which can promote caspase-independent death. Interestingly, NCO-01/04 increased the LC3-II-enriched protein fraction, indicating autophagosome accumulation as well as autophagy. Thus, NCO-01/04 simultaneously caused caspase activation and autophagy. These results suggest that NCO-01/04 is highly effective against ATL cells in caspase-dependent or -independent manners with autophagy, and that its clinical application might improve the prognosis of patients with this fatal disease.

Adult T-cell leukaemia/lymphoma (ATL) is a leukaemia derived from mature CD4^+^ T-cells with a poor prognosis, and develops after long-term infection with human T-cell leukaemia virus (HTLV)-1^1^,^2^,^3^. Host genetic and epigenetic abnormalities and host immunological status should be considered in attempts to understand the mechanism for the oncogenesis of ATL, although the underlying mechanisms of leukaemogenesis have not been fully elucidated^4^,^5^,^6^,^7^. Despite recent advances in chemotherapy, allogeneic hematopoietic stem cell transplantation, and supportive care, the prognosis for patients with ATL is one of the poorest among the haematological malignancies, with a 3-year overall survival rate of only 24% for the more aggressive subtypes of ATL^8^,^9^,^10^. Therefore, new strategies for therapy and prophylaxis of ATL, vaccines, and novel molecular targeted agents are still required^7^,^11^,^12^.

SIRT1 is a nicotinamide adenine dinucleotide^+^ -dependent deacetylase that counteracts multiple disease states associated with aging and may underlie some of the health benefits of calorie restriction^13^. SIRT1 plays crucial roles in a variety of physiological processes, including metabolism, apoptosis, and aging, through its ability to deacetylate numerous substrates, such as histones, p53, and NF-κB^14^. SIRT1 is regarded as a tumour promoter because of its increased expression in glioblastoma, prostate cancer, and primary colon cancer, and its function for inactivating proteins that are involved in tumour suppression and DNA damage repair^15^. Lack of SIRT1 expression increased the apoptosis of HTLV-1-infected cell lines, suggesting that SIRT1 acts as a tumour promoter in leukaemic cell lines^16^,^17^. Conversely, both breast cancer and hepatic cell carcinoma exhibit reduced SIRT1 levels compared with normal tissues, suggesting SIRT1 could act as tumour suppressor^18^. Taken together, these results indicate that SIRT1 could act as either a tumour promoter or tumour suppressor, depending on the cellular context or its targets in specific signalling pathways or specific cancers. However, the precise mechanisms underlying these contradictory activities are not well understood.

We previously reported that SIRT1 expression was significantly higher in ATL patients, especially acute ATL patients, than in healthy controls^16^,^17^. We further reported that sirtinol, a SIRT1 inhibitor, induced apoptosis via caspase family activation in leukaemic cell lines, especially HTLV-1-infected cell lines. These striking results added a new dimension for the development of SIRT1 inhibitors for leukaemia therapy. We previously designed and synthesized a series of 2-anilinobenzamide derivatives with SIRT1-inhibitory activity. Among these, NCO-01 and NCO-04 inhibited SIRT1 activity in enzyme assays and suppressed the growth of Daudi and HCT116 cells^19^.

In this study, we set out to assess the actions of these small-molecule inhibitors of SIRT1 in primary ATL cells and leukaemic cell lines. We found that NCO-01/04 induced apoptotic cell death with caspase activation in leukaemic cell lines, and also induced caspase-independent cell death with accumulation of endonuclease G in the nucleus and an LC3-II level, indicating autophagosome accumulation as well as autophagic type II cell death. This is the first evidence to demonstrate the cell growth-inhibitory effect of SIRT1 inhibitors with caspase-dependent or -independent cell death and autophagy in leukaemic cells.

## Results

### NCO-01/04 inhibit the viability of cells from ATL patients by inducing apoptosis

**Figure 1:**
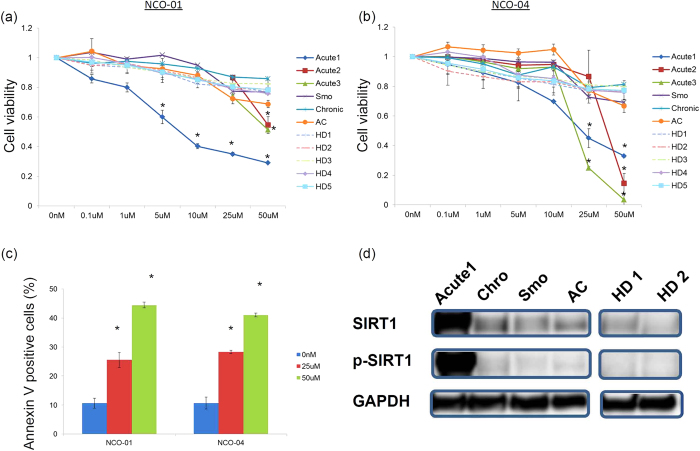
Effect of NCO-01/04 on cell viavility and Annexin V-positive cells in PBMCs. PBMCs were incubated at 2 × 10^5^ cells/mL in the presence of various concentrations of NCO-01 and NCO-04 (a–c). The viabilities of the cultured cells after 96 h were measured by WST-8 assays (**a**,**b**). Cells cultured in the absence of each SIRT1 inhibitor were assigned a relative viability of 1. Annexin V-positive cells cultured for 48 h were detected by flow cytometry (**c**). Data are means ± SD from three independent experiments. *P < 0.05 vs. 0 μM. Representative data for SIRT1 protein analysed by western blotting (**d**).

In the first set of experiments, we examined whether the novel small-molecule SIRT1 inhibitors NCO-01/04 affected the viability of peripheral blood mononuclear cells (PBMCs) from ATL patients (acute ATL, chronic ATL, and smouldering ATL), an asymptomatic HTLV-1 carrier (AC), and healthy donors (HDs). Fresh PBMCs from the acute ATL patients were more sensitive to NCO-01/04 than control PBMCs from the HDs (Fig. 1a,b). NCO-01 and NCO-04 showed potent activities with average GI_50_ values of 37.3 and 24.3 μM toward PBMCs from the acute ATL patients (Acute1−3), respectively.

To investigate whether the cell growth inhibition occurred through enhanced apoptotic cells in PBMCs from the acute ATL patient, the cells were treated with 25 or 50 μM NCO-01/04 and probed with Annexin V (Fig. 1c). The percentages of specific apoptotic cells with 50 μM NCO-01 were 38.0% (acute ATL), 5.7% (smouldering ATL), 12.0% (chronic ATL), and 9.9% (AC). The percentages of specific apoptotic cells with 50 μM NCO-04 were 34.0% (acute ATL), 6.0% (smouldering ATL), 11.0% (chronic ATL), and 10.9% (AC). NCO-01/04 treatment resulted in significant increases in apoptotic primary acute ATL cells. The SIRT1 protein levels were higher in primary ATL cells from the acute ATL patients than in those from the chronic ATL patients, smouldering ATL patients, AC, and HDs (Fig. 1d). The most sensitive was the acute ATL patient (Acute 1) with 98.5% ATL cells, while the Acute2, Acute3, smouldering and chronic ATL patients had 30.5%, 76.3%, 46.0%, and 1.0% ATL cells, respectively. The percentages of malignant cells (blasts) within the PBMCs were 98.9% (Acute 1), 32.1% (Acute 2), 88.4% (Acute 3), 81.7% (chronic), and 1.8% (smouldering).

### NCO-01/04 inhibits the cell viability of leukaemic cell lines

**Figure 2:**
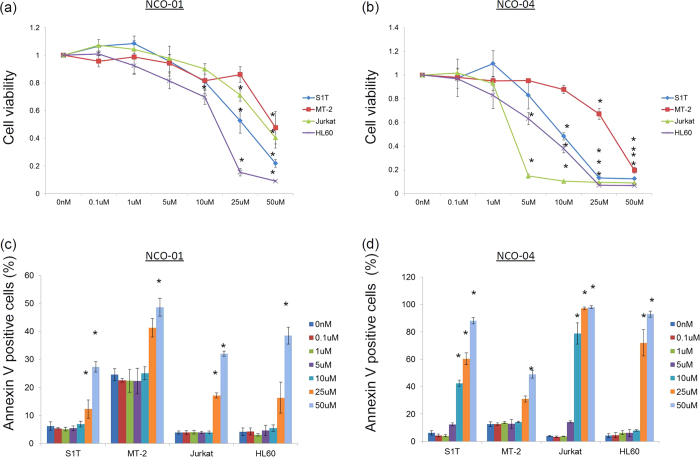
Effect of NCO-01/04 on cell viavility and Annexin V-positive cells in leukaemic cell lines. Cell lines were incubated at 2 × 10^5^ cells/mL in the presence of various concentrations of NCO-01 and NCO-04 for 72 h (**a**–**d**). The viabilities of the cultured cells were measured by WST-8 assays (**a**,**b**). Cells cultured in the absence of each SIRT1 inhibitor were assigned a relative viability of 1. Annexin V-positive cells were detected by flow cytometry (**c**,**d**). Data represent the mean percentages ± SD of apoptotic cells from three independent experiments. *P < 0.05 vs. 0 μM.

Next, we examined whether NCO-01/04 affected the cell viabilities of S1T, MT-2, Jurkat, and HL60 cells using water-soluble tetrazolium (WST)-8 assays. NCO-01/04 inhibited the growth of all four cell lines in a dose-dependent manner (Fig. 2a,b). NCO-01 showed potent activities with GI_50_ values of 36.1, 44.8, 41.3, and 9.9 μM for S1T, MT-2, Jurkat, and HL60 cells, respectively. NCO-04 showed potent activities with GI_50_ values of 11.6, 27.9, 2.6, and 8.7 μM, for S1T, MT-2, Jurkat, and HL60 cells, respectively.

### NCO-01/04 induces apoptosis with caspase activation in leukaemic cell lines

To examine whether the cell death induced by NCO-01/04 was apoptosis, we analysed the NCO-01/04-induced cell death by Annexin V and TUNEL staining. We observed that NCO-01/04 induced Annexin V-positive cells in the leukaemic cell lines (Fig. 2c,d). The percentages of specific Annexin V-positive cells with 50 μM NCO-01 were 22.6%, 15.0%, 10.4%, and 25.4% for S1T, MT-2, Jurkat, and HL60 cells, respectively. The percentages of specific Annexin V-positive cells with 50 μM NCO-04 were 87.3%, 18.0%, 97.9%, and 92.7% for S1T, MT-2, Jurkat, and HL60 cells, respectively.

**Figure 3:**
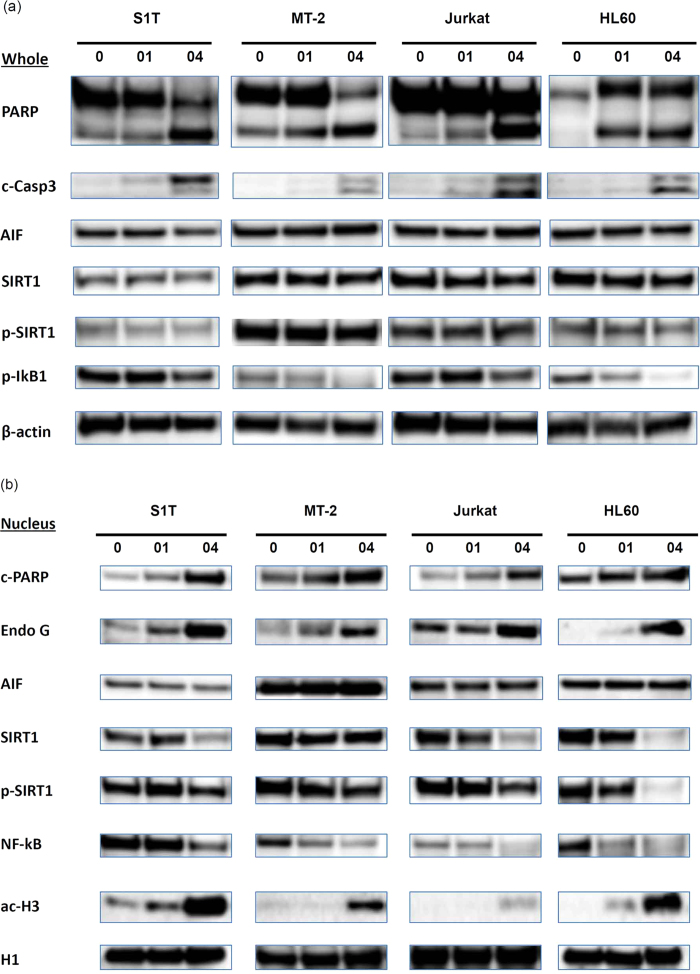
Effects of NCO-01/04 on apoptosis, CICD-related proteins, and SIRT1 protein in leukaemic cell lines. S1T, MT-2, Jurkat, and HL60 cells were treated with NCO-01 (50 μM) or NCO-04 (S1T and HL-60, 25 μM; MT-2, 50 μM; Jurkat, 10 μM) for 48 h. The protein levels in whole cell extracts were detected by western blotting with antibodies against each protein as indicated (a). Nuclear extracts from the treated cells were immunoblotted with specific antibodies (b).

To clarify the molecular mechanism underlying the NCO-01/04-induced apoptosis of S1T, MT-2, Jurkat, and HL60 cells, we examined the expression levels of several intracellular regulators of apoptosis in whole cells (Fig. 3a) or nuclear extracts (Fig. 3b) by western blotting. NCO-01/04 treatment cleaved PARP (c-PARP) and caspase-3 (c-Casp3), indicating that these cysteine proteases were involved in the regulation of apoptosis in the leukaemic cell lines, especially in NCO-04-treated cells. The cleavage of caspase 3 induced by NCO-01 was slightly decreased compared with NCO-04 treatment, however, the caspase activities (pan-caspase, caspase-3, 8, and 9) assessed by fluorochrome-labelled inhibitors of caspases (FLICA) were increased by NCO-01 as shown below. FLICA is a preferable marker for the detection of early phase apoptosis and more accurate for quantification of apoptotic cells^20^. A small number of bands identified using caspase-specific antibodies for western blotting could also be detected using the FLICA labelling methodology^21^.

### NCO-01/04 modulates SIRT1 protein and acetylation of histone

SIRT1 phosphorylation increases its substrate-binding affinity and deacetylase activity^22^. The SIRT1 and p-SIRT1 protein levels were stable in whole lysates of NCO-01/04-treated S1T, MT-2, Jurkat, and HL60 cells (Fig. 3a). Conversely, the SIRT1 and p-SIRT1 protein levels were reduced in the nucleus, especially in NCO-04-treated cells (Fig. 3b). Simultaneously, NCO-01/04 increased acetylation of histone H3, especially in NCO-04-treated cells.

The degradation of IκB and subsequent release of NF-κB require prior phosphorylation^23^. NCO-01/04 treatment decreased the phospho-IκB protein levels in the cytoplasm (Fig. 3a). Concomitantly, the NF-κB levels in the nucleus were markedly decreased (Fig. 3b), indicating that translocation of NF-κB from the cytoplasm to the nucleus was inhibited under conditions of NCO-01/04-induced cell death.

### NCO-01/04 induces loss of mitochondrial transmembrane potential and generation of Reactive oxygen species (ROS)

**Figure 4:**
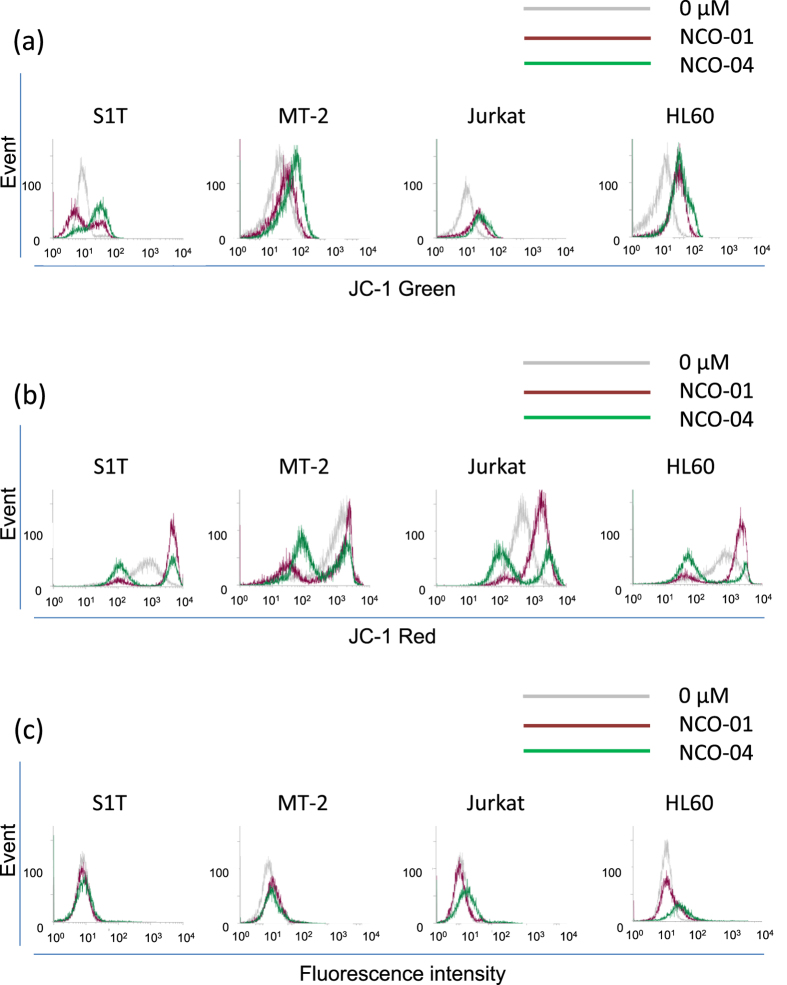
Effects of NCO-01/04 on the mitochondrial membrane potential and ROS formation. S1T, MT-2, Jurkat, and HL60 cells were treated with NCO-01 (50 μM) or NCO-04 (S1T and HL-60, 25 μM; MT-2, 50 μM; Jurkat, 10 μM) for 48 h (**a**,**b**). The cells were analysed for their green (**a**) and red (**b**) fluorescence emission components by flow cytometry. The ROS levels were determined by assaying the fluorescent product 5(6)-carboxy-2′,7′-dichlorofluorescein in viable cells by flow cytometry (**c**). Three independent experiments per cell line were performed, and representative results are presented.

In apoptosis, several key events occur in mitochondria, including the release of caspase activators, such as cytochrome c, and loss of mitochondrial transmembrane potential^24^. In healthy cells with high mitochondrial transmembrane potential, JC-1 spontaneously forms complexes showing intense red fluorescence. Conversely, in apoptotic cells with low mitochondrial transmembrane potential, JC-1 remains in its monomeric form and shows green fluorescence. By measuring the shift in fluorescence emission by flow cytometry, mitochondrial polarization was readily detected in NCO-01/04-treated cells (Fig. 4a,b). Notably, the majority of NCO-01/04-treated cells showed green fluorescence reflecting low mitochondrial transmembrane potential among the leukaemic cell lines.

The proteins released from the mitochondrial intermembrane space exert multifaceted effects, ranging from caspase activation to chromatin condensation, DNA strand breakage, and generation of ROS^25^. To clarify the effects of NCO-01/04 on the intracellular redox status, we determined the ROS levels by measuring the oxidation of non-fluorescent carboxy-H_2_DCFDA to the highly fluorescent 5(6)-carboxy-2′,7′-dichlorofluorescein. As shown in Fig. 4c, NCO-04 stimulated ROS formation in Jurkat and HL60 cells, while intracellular ROS accumulation was not detected in NCO-01-treated leukaemic cell lines and NCO-04-treated S1T and MT-2 cells.

### NCO-01/04 induces both caspase-dependent and -independent cell death

**Figure 5:**
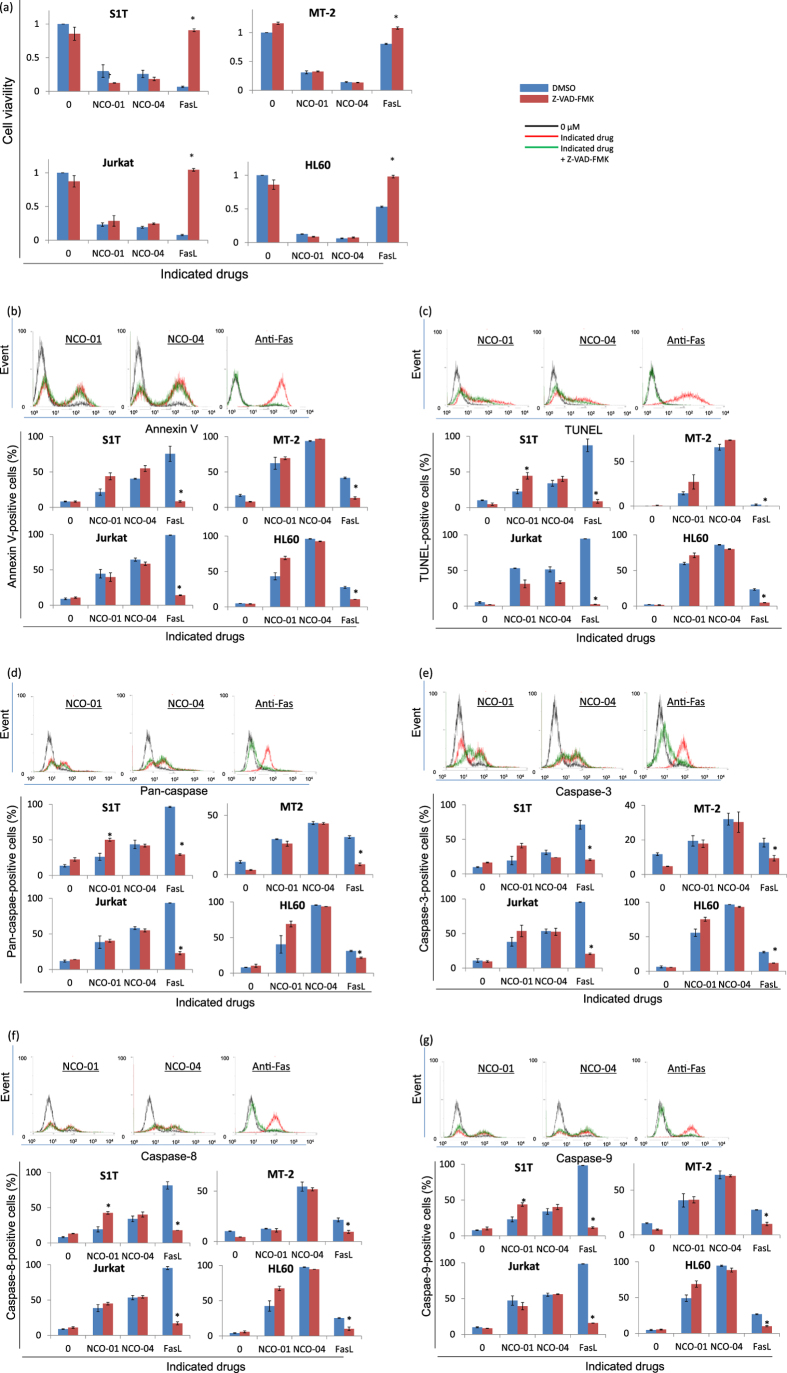
Effects of a pan-caspase inhibitor on NCO-01/04-induced cell death. S1T, MT-2, Jurkat, and HL60 cells were treated with NCO-01 (50 μM) or NCO-04 (S1T and HL-60, 25 μM; MT-2, 50 μM; Jurkat, 10 μM), anti-Fas antibody (100 ng/mL) and Z-VAD-FMK (40 μM) for 72 h (**a**–**g**). The viabilities of the cultured cells were measured by WST-8 assays (**a**). Annexin V-positive, TUNEL-positive, and caspase-positive cells were detected by flow cytometry (**b**–**g**). Representative data for Jurkat cells are indicated in the upper panels in panels B–G. Data represent the mean percentages ± SD from three independent experiments. *P < 0.05 vs. each reagent in the absence of Z-VAD-FMK.

Next, we assessed the effects of a pan-caspase inhibitor on NCO-01/04-induced cell death (Fig. 5). NCO-01/04 induced significant growth inhibition with Annexin V-positive cells and DNA fragmentation in leukaemic cell lines. FLICA probes have been consistently shown to be highly reliable reporters of caspase activation and convenient markers of apoptotic cells^20^. NCO-01/04 also activated caspase activity (pan-caspase, caspase-3, caspase-8, and caspase-9). However, the pan-caspase inhibitor Z-VAD-FMK did not inhibit the cell death, Annexin V-positive cells, or DNA fragmentation, but did suppress Fas-mediated cell death (Fig. 5a–c). Conversely, Z-VAD-FMK did not inhibit the pan-caspase, caspase-3, caspase-8, and caspase-9 activities, but did suppress the Fas-mediated caspase activity (Fig. 5d–g). When other signs of cell death (such as mitochondrial dysfunction, phosphatidylserine exposure, or plasma membrane permeabilisation) are considered, caspase inhibition frequently does not confer cytoprotection. The ideal inhibitor with optimal pharmacokinetic and pharmacodynamics properties might still be lacking. Therefore, uninhibited caspases or caspases activated by cell death were sufficient to trigger a lethal effector mechanism.

Some of the mitochondrial proteins (apoptosis-inducing factor: AIF; endonuclease G) released as a result of mitochondrial outer membrane permeabilisation (MOMP), which leads to release of pro-apoptotic proteins from the mitochondrial intermembrane space, can promote caspase-independent death (CICD) through mechanisms that are relatively poorly defined^26^. NCO-01/04 treatment increased the protein levels of endonuclease G in the nucleus, especially in NCO-04-treated cells (Fig. 3b). Conversely, the AIF protein levels in the nucleus and whole cells were stable in NCO-01/04-treated S1T, MT-2, Jurkat, and HL60 cells (Fig. 3).

### NCO-01/04 induce autophagy in leukaemic cell lines

**Figure 6:**
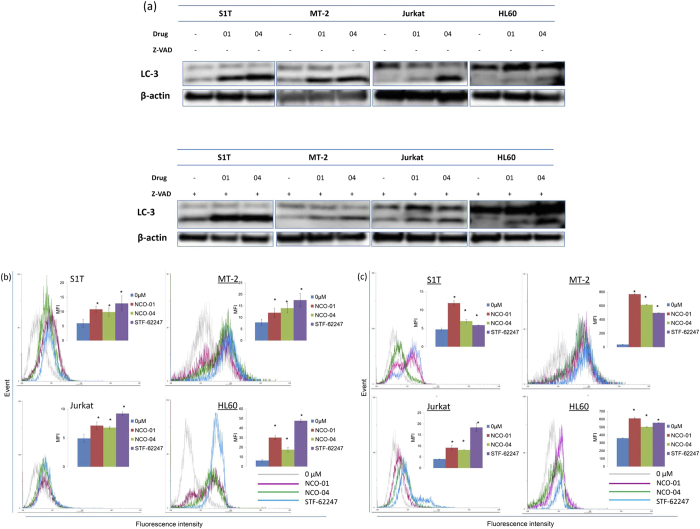
Effects of NCO-01/04 on autophagy in leukaemic cell lines. S1T, MT-2, Jurkat, and HL60 cells were treated with NCO-01 (50 μM) or NCO-04 (S1T and HL-60, 25 μM; MT-2, 50 μM; Jurkat, 10 μM) and Z-VAD-FMK (40 μM) for 48 h (**a**). Protein levels were detected by western blotting with antibodies against each protein as indicated. S1T, MT-2, Jurkat, and HL60 cells were treated with NCO-01 (50 μM) or NCO-04 (S1T and HL-60, 25 μM; MT-2, 50 μM; Jurkat, 10 μM) and STF-62247 (10 μM) for 72 h (**b**,**c**). Cellular autophagic flux was evaluated using the FlowCellect™ Autophagy LC3 Antibody-based Assay Kit (**b**) and Cyto-ID autophagy detection kit (**c**). Cells were incubated for 30 min with the autophagy flux inhibitor provided (**b**) or for 2 h with bafilomycin A1 (**c**). Data represent the mean percentages ± SD of autophagic cells from three independent experiments per cell line. Representative results are presented. X-axis in each bar graph: Mean Fluorescence Intensity (MFI). *P < 0.05 vs. 0 μM.

In a number of different models, caspase inhibition does not maintain cellular viability and instead shifts the morphology of death from apoptosis to non-apoptotic pathways^27^. Autophagy can degrade cellular components, such that cells eventually activate the apoptosis machinery. Conversion of the soluble form LC3-I to the autophagic vesicle-associated form LC3-II is considered to be a specific marker for autophagosome promotion. NCO-01/04 significantly increased the levels of LC3-II (lipidated LC3) in the presence or absence of Z-VAD-FMK (Fig. 6a).

Monitoring the translocation of LC3 using flow cytometry allows discrimination between cytosolic- and autophagosome-associated populations. The use of an autophagy inhibitor will prevent the lysosomal degradation of LC3, allowing for quantification of its fluorescence. Autophagy was measured using flow cytometry by quantifying LC3-II mean fluorescence intensity using the FlowCellect Autophagy LC3 Antibody-based Assay kit. Use of this kit includes a step where cytosolic LC3-I is washed from the cell, leaving only membrane bound LC3-II prior to staining. To assess autophagic flux before LC3 detection with anti-LC3 antibody, cells were incubated for 30 min with the autophagy flux inhibitor provided by the specified kit. Autophagy levels are increased in presence of NCO-01, NCO-04, and the STF-62247 autophagy inducer (Fig. 6b, Supplemental Figure 1).

Autophagy detection was also performed using the CytoID Autophagy detection kit. CytoID Green autophagy dye was validated by observing co-localization of the dye and RFP-LC3 in HeLa cells using fluorescence microscopy^28^. Autophagy levels are also increased in the presence of NCO-01, NCO-04, and STF-62247 pre-treated with bafilomycin A1, a specific inhibitor of vacuolar proton ATPase, whose inhibition is known to block the fusion of autophagosomes with lysosomes for 2 h (Fig. 6c).

To investigate the effects of SIRT1 protein on autophagy in MT-2 and Jurkat cells, we analysed the LC3 protein levels in these cells after SIRT1 knockdown (Supplemental Figure 2). We confirmed that the small interfering RNA (siRNA) against SIRT1 specifically knocked down SIRT1 expression in MT-2 and Jurkat cells. Lack of SIRT1 expression did not influence the levels of LC3-II, p62, and beclin 1, which are involved in autophagosome formation^25^.

### NCO-01/04-induced cell death is dependent on SIRT1 protein

**Figure 7:**
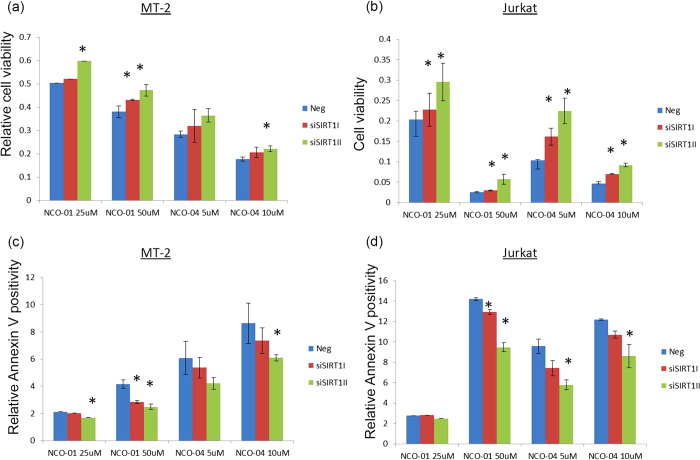
Effects of SIRT1 silencing on NCO-01/04 induced cell death. MT-2 and Jurkat cells were transfected with a negative control siRNA (Neg) or siRNA against SIRT1 (siSIRT1I and siSIRT1II) and treated with NCO-01 or NCO-04 for 48 h (**a**–**d**). The viabilities of the cultured cells were measured by WST-8 assays (**a**,**b**). Apoptotic cells were detected by Annexin V staining using flow cytometry (**c**,**d**). Cells cultured in the absence of each SIRT1 inhibitor in the transfected condition were assigned a relative viability and Annexin V positivity of 1. Data represent the mean percentages ± SD from three independent experiments. *P < 0.05 vs. Neg.

To evaluate the relevance of the SIRT1-mediated effects on cell death induced by NCO-01/04, MT-2 and Jurkat cells with SIRT1 knockdown were treated with NCO-01/04. Cells cultured in the absence of the inhibitors under each transfected condition were assigned a relative viability of 1 (Fig. 7). NCO-01/04 treatment decreased the cell survival rate; however, cell viability in the SIRT1-knockdown groups with NCO-01/04 treatment was higher than in the SIRT1-expressing groups with NCO-01/04 treatment. Thus, this effect was attenuated under SIRT1 knockdown conditions, suggesting that NCO-01/04 could target the SIRT1 protein. NCO-01/04 treatment also increased Annexin V-positive cells, while SIRT1-knockdown cells showed decreased Annexin V-positivity induced by NCO-01/04 treatment compared with SIRT1-expressing cells. Therefore, SIRT1 knockdown could attenuate, but could not completely reverse, the NCO-01/04-induced cell death.

## Discussion

SIRT1, a nicotinamide adenine dinucleotide^+^ -dependent deacetylase, protects cells against stress-induced apoptosis^29^. SIRT1 is also consistently upregulated in malignant cells or tissues from patients with glioblastoma, and prostate, colorectal, or skin cancer^30^. Casein kinase II-mediated phosphorylation increases the ability of SIRT1 to deacetylate p53 and protect cells against apoptosis after DNA damage^31^. Previously, we showed that the SIRT1 and p-SIRT1 protein levels were higher in primary acute ATL cells than in healthy PBMCs^17^. In the present study, the SIRT1 expression levels were higher in primary ATL cells from an acute ATL patient than in cells from a chronic ATL patient, smouldering ATL patient, AC, and HDs. Consequently, we examined how NCO-01 and NCO-04, which are novel small-molecule SIRT1 inhibitors, regulate cell death pathways in primary ATL cells. Our results showed that NCO-01/04 inhibited the cell viability and induced apoptosis in primary ATL cells, with greater selectivity for acute ATL cells with high SIRT1 protein expression. NCO-01/04 also reduced the cell viability with loss of p-SIRT1 in leukaemic cell lines. Thus, methods to block the function of SIRT1 may be useful for the development of ideal therapeutic agents for leukaemia, especially in patients with ATL.

In our previous report on a series of 2-anilinobenzamide derivatives, NCO-01 and NCO-04 inhibited SIRT1 activity with IC_50_ values of 58 and 52 μM in enzyme assays, respectively^19^. Furthermore, NCO-01 and NCO-04 suppressed the growth of HCT116 cells with GI_50_ values of 50 and 43 μM, and the growth of Daudi cells with GI_50_ values of 31 and 26 μM, respectively. Here, we demonstrated that NCO-01/04 inhibited the growth of leukaemic cell lines in dose-dependent manners with Annexin V-positive cells and DNA fragmentation. SIRT1 knockdown could also attenuate the NCO-01/04-induced cell death. These results indicated NCO-01/04 induced cell death by apoptosis in leukaemic cell lines via the inhibition of SIRT1 activity.

SIRT1 could act as either an apoptosis promoter or an apoptosis suppressor, depending on the cellular context or its targets in specific signalling pathways^18^. However, the precise mechanisms underlying these contradictory activities are not well understood. Interestingly, a lack of SIRT1 expression increased the apoptosis of HTLV-1-infected cell lines^16^,^17^. In this study, the lack of SIRT1 expression increased the Annexin V-positive cells (data not shown: specific apoptotic cells in MT-2: 6.5% in siSIRT1I, 8.7% in siSIRT1 II; specific apoptotic cells in Jurkat: 4.3% in siSIRT1I, 5.5% in siSIRT1 II). NCO-01/04 also induced an increase in the Annexin V-positive cells in the SIRT1-knockdown cells. Therefore, a lack of the target for NCO-01/04 could not induce SIRT1-dependent cell death before the further decrease of SIRT1 activity rescued the effect of a SIRT1 inhibitor. Additionally, the effect of NCO-01/04 cannot be fully reversed by SIRT1 knockdown, indicating that NCO-01/04-induced cell death has SIRT1 protein-dependent and -independent pathways. NCO-01 and NCO-04 also inhibited SIRT2 activity in enzyme assays with IC_50_ values of 25 and 33 μM, respectively. SIRT2 is a tumour suppressor gene with an essential role in maintaining the integrity of mitosis, and SIRT2 reductions can also induce apoptosis of HeLa cells by affecting the levels of p53^32^. SIRT2 inhibitors exhibit anti-tumour effects by promoting apoptosis and inhibiting cell growth in cancer^33^. Thus, NCO-01/04-induced cell death has not only SIRT1 protein-dependent but also SIRT2 protein-dependent pathway. Conversely, the effects of NCO-04 with cell growth inhibition, apoptosis induction, and caspase activation were greater than those of NCO-01. NCO-04 significantly enhanced the acetylation of histone H3 in leukaemic cell lines, while NCO-01 did not enhance histone H3 acetylation in MT-2 and Jurkat cells. These results suggest that the substrates differ between NCO-01 and NCO-04.

Apoptosis is formally defined by the cell morphology, in which the cell shrinks, shows nuclear condensation, and often becomes fragmented into smaller membrane-bound bodies, although more recent descriptions have relied on the detection of caspase activation^25^. Although caspases are evolutionarily conserved, it seems that the equation of “apoptosis = caspase activation” does not apply to mammals for several reasons^27^. For example, some cells succumb to developmental cell death in a caspase-independent manner, indicating that caspase activation does not necessarily lead to apoptosis^34^. CICD is correlated with a progressive decline in mitochondrial function and ATP generation that precedes mitochondrial release of AIF and endonuclease G, suggesting that MOMP contributes to CICD primarily through loss of mitochondrial function^26^. Following MOMP, the mitochondrial membrane potential is dissipated in both caspase-dependent and -independent manners. In this study, NCO-01/04 treatment induced pan-caspase, caspase-3, caspase-8, and caspase-9 activities, indicating that these cysteine proteases were involved in the regulation of apoptosis in the leukaemic cell lines. NCO-01/04 also enhanced the endonuclease G level in the nucleus with loss of the mitochondrial transmembrane potential. Interestingly, the pan-caspase inhibitor Z-VAD-FMK did not inhibit this caspase-dependent cell death. DNA fragmentation also occurred in the presence of both NCO-01/04 and the caspase inhibitor, indicating that NCO-01/04 simultaneously induced caspase-dependent and -independent cell death in the leukaemic cell lines. These results suggest that the molecules involved in the process of caspase-independent DNA fragmentation may augment the caspase activity, such as secondary caspase activation. Thus, caspase-independent cell death induces caspase activation through an unknown mechanism. Although there is no doubt that massive caspase activation is sufficient for the induction of cell death, inhibitor experiments indicate that caspase inhibition in mammalian cells is often insufficient to avoid cell death.

Autophagy, a lysosomal pathway involving bulk degradation of cytoplasmic contents, has been identified as a prime suspect in such death, and recent studies have implicated the autophagy pathway as a cause of non-apoptotic cellular demise^35^. The term “autophagic cell death” describes a form of programmed cell death that is morphologically distinct from apoptosis and presumed to result from excessive levels of cellular autophagy. MOMP triggers the removal of permeabilized mitochondria by the autophagic machinery^26^. When inhibition of caspases does not inhibit cell death, it often leads to a shift in the morphology of cell death, from the appearance of classical apoptosis to the occurrence of “apoptosis-like” cell death, autophagic cell death, or even necrosis^27^. Sirtinol, a SIRT1 inhibitor, induced autophagy with increased LC3-II expression, while caspase activity was not altered in MCF-7 human breast cancer cells^36^. We examined the possibility that NCO-01/04-induced cell death occurs through the autophagic machinery. NCO-01/04 increased the LC3-II levels with loss of mitochondrial transmembrane potential and caspase activation. A caspase inhibitor had no effect on NCO-01/04-induced cell death with caspase activation and autophagy induction. These results indicate that NCO-01/04 induced cell death effectively, causing caspase activation and autophagy, although it is not yet known whether this caspase activation requires autophagic type II cell death.

In conclusion, the novel SIRT1 inhibitors NCO-01/04 induced growth inhibition and apoptosis in cells from ATL patients. NCO-01/04 also induced caspase-dependent or -independent cell death and autophagic cell death in leukaemic cell lines. Thus, NCO-01/04 simultaneously induce apoptosis and autophagy. Although apoptosis and autophagy share many common mechanisms, the current knowledge on molecular interactions between the autophagic and apoptotic pathways is incomplete and fragmented. Therefore, it may be necessary to further elucidate the relationship between apoptosis and autophagy following NCO-01/04 treatment in fresh leukaemic cells. Our results evoke hope that a strategy for cancer treatment may be developed based on SIRT1 inhibitors.

## Materials and Methods

### Clinical samples

Methods used in this study were carried out in accordance with the approved guidelines by the Committees for Ethical Review of Research involving Human Subjects at Kagoshima University. All subjects provided written informed consent for participation in this study and a review of their medical records, and provided a sample of peripheral blood for isolation of PBMCs. The subjects evaluated in this study were three acute type ATL patients (median age 64 years, range 52–77, one male and two females), one chronic type ATL patient (67 years, female), one smouldering type ATL patient (79 years, female), one AC (66 years, female), and five HDs (median age 36.0 years, range 30–42, all males). The ATL patients and AC came to the hospital for examination of HTLV-1 infections and clinical check-up. The subjects were examined by standard serological testing for the presence of HTLV-1 and by haematological/Southern blotting analysis for diagnosis of ATL. A subject seropositive for HTLV-1 without clinical symptoms of HTLV-1-related diseases was designated the AC. The classification of ATL was performed according to the criteria of Shimoyama^37^. Blood (20 ml) was taken from subjects. PBMCs were separated from the peripheral blood samples by Ficoll/Hypaque (Pharmacia, Uppsala, Sweden) density gradient centrifugation at 400 × *g* for 30 min. Freshly isolated PBMCs were used for western blotting and apoptosis analyses. The remaining PBMCs were cryopreserved in liquid nitrogen until further examination as described previously^38^,^39^. Previous studies^16^,^17^ have suggested the concordance of protein and gene expression in fresh and cryopreserved ATL cells from individual patients; nevertheless, we validated important observations in freshly isolated ATL cells.

### Cell lines

The cell lines S1T (HTLV-1-infected CD4^+^ T-cell line derived from an ATL patient)^40^, MT-2 (HTLV-1-infected T-cell line derived from normal human leukocytes transformed by leukaemic T-cells from an ATL patient)^41^, Jurkat (HTLV-1-uninfected T-cell line), and HL60 (acute myeloid leukaemia cell line) were cultured in RPMI-1640 medium supplemented with 100 U/mL penicillin, 0.1 mg/mL streptomycin, 2 mM l-glutamine, and 10% heat-inactivated foetal calf serum.

### Reagents

The novel SIRT1 inhibitors evaluated in this study, NCO-01 and NCO-04, were described in a previous report^19^. Anti-Fas monoclonal antibody CH11 and caspase inhibitor Z-VAD-FMK were purchased from Medical and Biological Laboratories (MBL, Nagoya, Japan). STF-62247 was purchased from Merck Millipore (Darmstadt, Germany). Bafilomycin A1 was purchased from Adipogen (Epalinges, Switzerland).

Primary antibodies against PARP, cleaved-PARP, NF-κB, p-IκB, endonuclease G, acetyl-histone H3, caspase-3, SIRT1, p-SIRT1 (Ser47), AIF, β-actin, histone H1, and GAPDH were purchased from Cell Signaling Technology (Beverly, CA, USA), and those against beclin 1, p62, and LC3 were obtained from MBL. Horseradish peroxidase-conjugated secondary antibodies were purchased from Vector Laboratories (Burlingame, CA, USA).

### Protein extraction and western blotting analysis

Whole-cell extracts were lysed in RIPA Lysis Buffer (Santa Cruz Biotechnology, Santa Cruz, CA, USA). Nuclear extracts were obtained using NE-PER Nuclear and Cytoplasmic Extraction Reagents (Pierce Biotechnology, Rockford, IL, USA), according to the manufacturer’s protocols. The whole-cell and nuclear extracts were used immediately or stored at −80 °C until analysis. Western blotting was performed as described previously^17^. Briefly, cell extracts were subjected to SDS-PAGE, electroblotted onto Immobilon-P membranes (Merck Millipore), and analysed for immunoreactivity with appropriate primary and secondary antibodies using Can Get Signal Solution (Toyobo, Osaka, Japan). The reaction products were visualized using Chemi-Lumi One Super (Nacalai Tesque, Kyoto, Japan), according to the manufacturer’s protocols. The band intensities in images were analysed using ChemiDoc™ XRS (Bio-Rad, Hercules, CA).

### Cell viability assay

The effects of NCO-01/04 on cell viability were examined using a cell proliferation reagent, WST-8 (Wako Chemicals, Osaka, Japan)^42^. Briefly, aliquots containing 2 × 10^5^ cells/mL (cell lines) or 1 × 10^6^ cells/mL (PBMCs) were incubated in 96-well plates in the absence or presence of NCO-01/04 for 72 or 96 h. WST-8 (10 μM) was added for the last 2 h of incubation, and the absorbances at 450 nm (A_450_) were measured using an Infinite 200 PRO (TECAN, Männedorf, Switzerland). Measurements of mitochondrial dehydrogenase cleavage of WST-8 to produce formazan dye were used to indicate the cell viabilities.

### Apoptosis analysis

PBMCs, and S1T, MT-2, Jurkat, and HL60 cells were treated with various concentrations of NCO-01/04 for specified periods. DNA fragmentation was detected by TUNEL assays using a MEBSTAIN Apoptosis Kit Direct (MBL). Apoptotic cells were detected by staining with Annexin V- fluorescein isothiocyanate (FITC) (MBL) and subjected to flow cytometry analysis using a Cell Analyzer EC800 (Sony, Tokyo, Japan)^11^,^43^. The percentages of specific apoptotic cells were calculated as follows: % specific apoptotic cells = (Annexin V-positive cells − spontaneous Annexin V-positive cells)/ (100 − spontaneous Annexin V-positive cells) × 100.

### Mitochondrial transmembrane potential assay

Measurements of mitochondrial transmembrane potential were performed using a JC-1 Mitochondrial Membrane Potential Assay Kit (Cayman Chemical Company, Ann Arbor, MI). Briefly, after cultured cells were incubated with JC-1 Staining Solution for 30 min, functional mitochondria containing red JC-1 J-aggregates and apoptotic or unhealthy cells with collapsed mitochondria containing green JC-1 monomers were analysed according to the manufacturer’s recommended protocol.

### ROS detection

The carboxy derivative of fluorescein, carboxy-H_2_DCFDA (C400), carries additional negative charges that improve its retention compared with non-carboxylated forms. Cultured cells were incubated with pre-warmed phosphate-buffered saline containing a final working concentration of 10 mM probe for 30 min, and then analysed by flow cytometry using the Cell Analyzer EC800.

### Detection of caspase activity

FLICA have been designed as affinity labels of the enzyme active centre of caspases^44^. Use of FLICA allows for a convenient estimation of apoptosis by both cytometry and fluorescence microscopy^45^. Caspase activity was assessed using a CaspTag Pan Caspase *In Situ* Assay Kit (Chemicon, Temecula, CA, USA), APOPCYTO Intracellular Caspase-3 or -8 Activity Detection Kit (MBL), and CaspGLOW Fluorescein Active Caspase-9 Staining Kit (BioVision, Milpitas, CA, USA), according to the manufacturers’ instructions. Briefly, 2 × 10^5^ cells were cultured for 60 min in the presence of the substrate, washed, and analysed by flow cytometry.

### Autophagy analysis by flow cytometry

Autophagy was evaluated using the FlowCellect™ Autophagy LC3 Antibody-based Assay kit (Merck Millipore) according to manufacturer’s instructions^46^. In brief, discrimination between cytosolic- and autophagosome associated LC3 is achieved by monitoring the translocation of LC3 using flow cytometry. Because autophagy is a constitutive cellular degradation process, pre-treatment with lysosomal inhibitor is required for 30 min prior to treatment of the 72-h-cultured sample with anti-LC3 FITC to prevent lysosomal degradation of LC3. Quantification of fluorescence can then be performed using anti-LC3 FITC. Cytosolic and autophagosomic populations are differentiated by washing cells to remove cytosolic LC3-I and retaining only membrane-bound LC3-II prior to staining.

Cellular autophagic flux (turnover) was also monitored using a Cyto-ID autophagy detection kit (Enzo Life Sciences, Farmingdale, NY, USA) according to the manufacturer’s instructions^28^,^47^. In brief, the 488-nm excitable Cyto-ID green autophagy detection reagent supplied with the kit becomes brightly fluorescent in vesicles produced during autophagy serving as a convenient tool to detect autophagy at a cellular level. Following treatment, the cells were washed in phosphate buffered saline, and resuspended in 1000× dilution of the detection reagent. After 30 min of incubation at 37 °C, the cells were washed and analysed by flow cytometry.

### siRNA

To silence SIRT1 expression, a pre-designed double-stranded Human SIRT1 Validated Stealth RNAi™ siRNA (Invitrogen, Carlsbad, CA, USA) was used. Stealth RNAi™ siRNA Negative Control LO GC (Invitrogen) was used as a negative control. MT-2 or Jurkat cells were transfected with each siRNA using a Neon Transfection System (Invitrogen) as described previously^48^.

### Statistical analysis

Data are expressed as means ± SD. For data analyses, two-tailed Student’s *t*-tests and Wilcoxon matched-pairs tests were performed using Excel 2010 (Microsoft Japan, Tokyo, Japan) and Statcel2 software (OMS Publishing Inc., Tokyo, Japan). In all tests, values of *P* < 0.05 were considered statistically significant.

## Additional Information

**How to cite this article**: Kozako, T. *et al.* Novel small-molecule SIRT1 inhibitors induce cell death in adult T-cell leukemia cells. *Sci. Rep.* **5**, 11345; doi: 10.1038/srep11345 (2015).

## Supplementary Material

Supplementary Information
