# Supplementary material for: Novel small-molecule SIRT1 inhibitors induce cell death in adult T-cell leukaemia cells
Source: Sci Rep. 2015 Jun 19;5:11345. doi: 10.1038/srep11345 (PMC4473680; doi:10.1038/srep11345)
Supplement: Supplementary Information [file srep11345-s1.pdf]

Supplementary Information

Novel small-molecule SIRT1 inhibitors induce cell death in adult T-cell leukemia cells

Tomohiro Kozako, Takayoshi Suzuki, Makoto Yoshimitsu, Yuichiro Uchida, Ayako Kuroki, Akiyoshi Aikawa, Shin-ichiro Honda, Naomichi Arima, Shinji Soeda

Novel small-molecule SIRT1 inhibitors induce cell death in adult T-cell leukemia cells
Kozako et al. Supplemental Figure 1

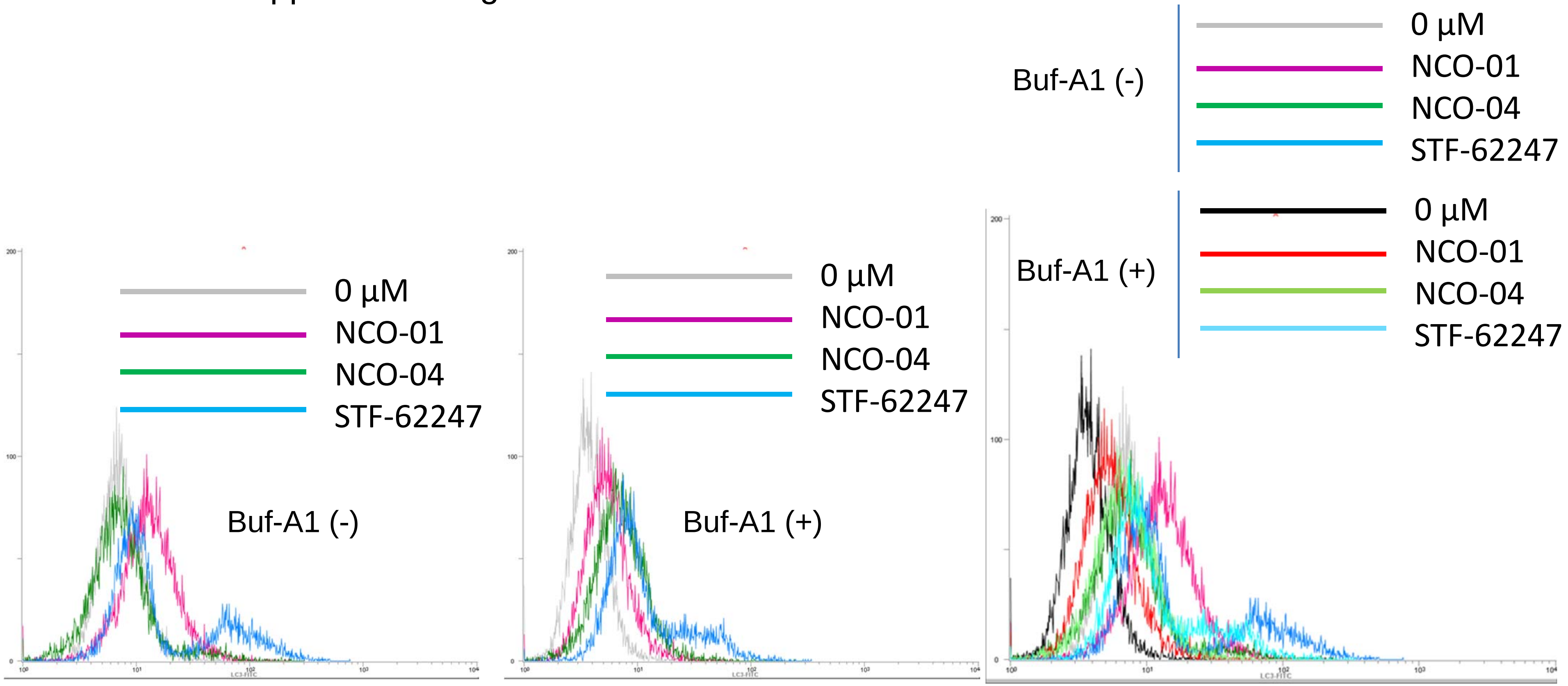

Supplemental Figure 1

Effects of NCO-01/04 on autophagy in leukaemic cell lines. Jurkat cells were treated with NCO-01 (50 μM) or NCO-04 (10 μM) and STF-62247 (10 μM) for 72 h. Cellular autophagic flux was evaluated using the Cyto-ID autophagy detection kit. Cells were incubated for 2 h with or without bafilomycin A1 (Buf-A1). S1T, MT-2, and HL60 cells were also treated with NCO-01 (50 μM) or NCO-04 (S1T and HL-60, 25 μM; MT-2, 50 μM) and STF-62247 (10 μM) for 72 h (data not shown). Three independent experiments per cell line were performed, and representative results are presented.

Novel small-molecule SIRT1 inhibitors induce cell death in adult T-cell leukemia cells
Kozako et al. Supplemental Figure 2

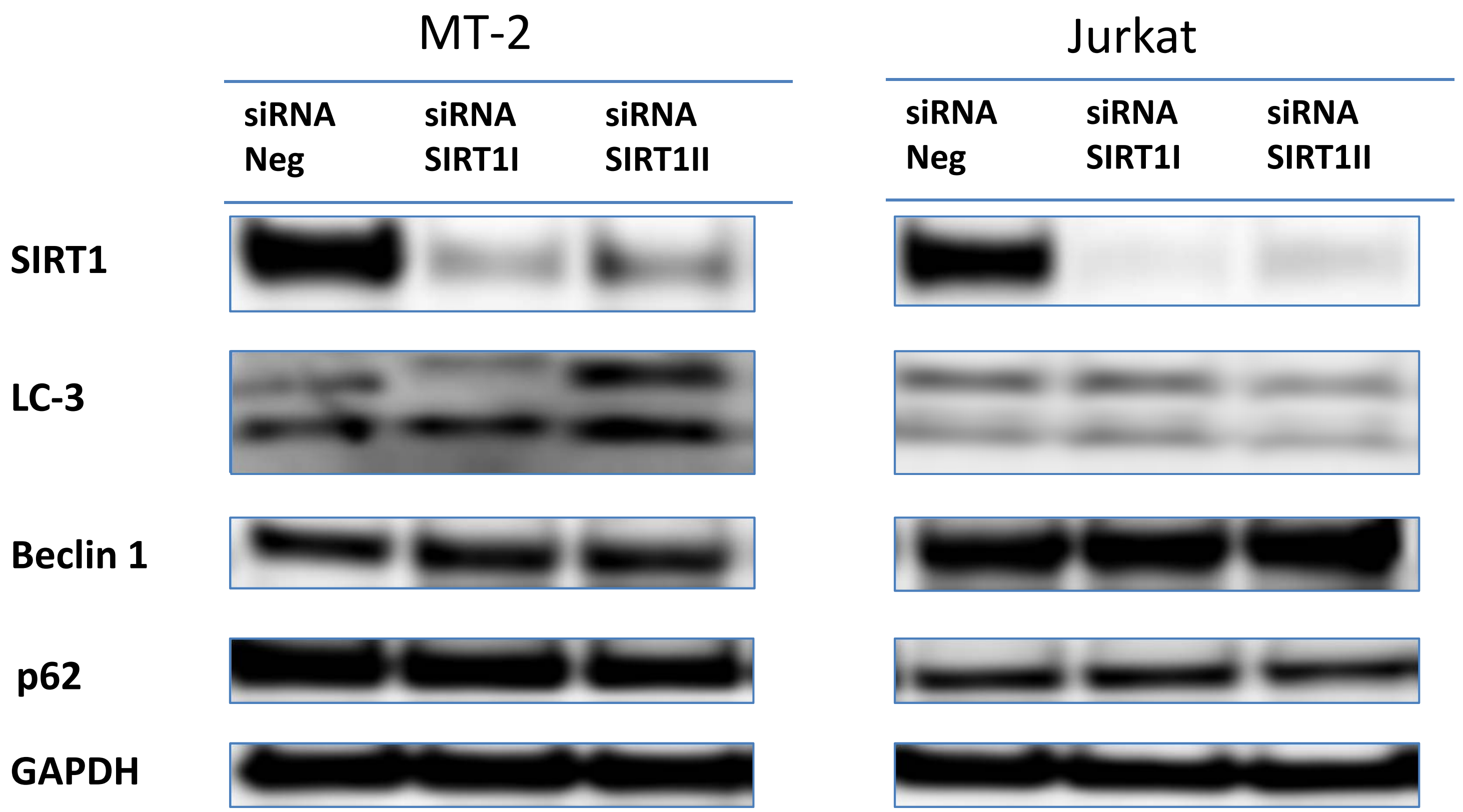

Supplemental Figure 2

Effects of SIRT1 silencing on autophagy-related proteins. MT-2 and Jurkat cells were transfected with a negative control siRNA (Neg) or siRNA against SIRT1 (siSIRT1I and siSIRT1II). Protein levels were detected by western blotting with antibodies against each protein as indicated. Each siRNA experiment was performed in triplicate, and representative results are presented.
